# Early spatiotemporal evolution of the immune response elicited by adenovirus serotype 26 vector vaccination in mice

**DOI:** 10.1128/jvi.00247-25

**Published:** 2025-03-31

**Authors:** Eryn Blass, Alessandro Colarusso, Malika Aid, Rafael A. Larocca, R. Keith Reeves, Dan H. Barouch

**Affiliations:** 1 Center for Virology and Vaccine Research, Beth Israel Deaconess Medical Center, Harvard Medical School 1811 , Boston, Massachusetts, USA; 2 Ragon Institute of MGH, MIT and Harvard 200750 , Cambridge, Massachusetts, USA; International Centre for Genetic Engineering and Biotechnology, Trieste, Italy

**Keywords:** immunization, adenoviruses, innate immunity

## Abstract

As the first responder to immunological challenges, the innate immune system shapes and regulates the ensuing adaptive immune response. Many clinical studies evaluating the role of innate immunity in initiating vaccine-elicited adaptive immune responses have largely been confined to blood due to the inherent difficulty in acquiring tissue samples. However, the absence of vaccine-site and draining lymph node information limits the understanding of early events induced by vaccination that could potentially shape vaccine-elicited immunity. We, therefore, utilized a mouse model to investigate the spatiotemporal evolution of the immune response within the first 24 hours following intramuscular adenovirus serotype 26 (Ad26) vector vaccination in tissues. We show that the Ad26 vaccine-elicited innate immune response commences by 1 hour and rapidly evolves in tissues and blood within the first 24 hours, as reflected by the detection of cytokines, chemokines, cellular responses, and transcriptomic pathways. Furthermore, serum levels of IL-6, MIG, MIP-1α, MIP-1β, and TNF-α at 6 hours post-vaccination correlated with the frequency of vaccine-elicited memory CD8^+^ T cell responses evaluated at 60 days post-vaccination in blood and tissues. Taken together, our data suggest that the immune response to Ad26 vector vaccination commences quickly in tissues by 1 hour and that events by as early as 6 hours post-vaccination can shape vaccine-elicited CD8^+^ T cell responses at later memory time points.

**IMPORTANCE:**

Prior studies have largely concentrated on innate immune activation in peripheral blood following vaccination. In this study, we report the detailed spatial and temporal innate immune activation in tissues following Ad26 vaccination in mice. We observed rapid innate activation not only in peripheral blood but also in draining lymph nodes and at the site of inoculation. Our findings provide a more detailed picture of the host response to vaccination than previously reported.

## INTRODUCTION

Innate immunity plays a critical role as an initial barrier to infection and forms an integral component in the initiation and development of adaptive immune responses. As the generation of protective adaptive immune responses is critical to the development of successful vaccines, understanding the bridge between innate and adaptive immunity provides greater insights into the immunological mechanisms of vaccination and how immune responses are ultimately tailored.

Adenovirus (Ad) vectors have been extensively studied for vaccine development for infectious diseases such as HIV (1), Zika (2), Ebola (3), and SARS-COV2 (4, 5). CD8^+^ T cell responses are strongly induced by Ad vectors, and as such, this vaccine platform has the utility of being used for T cell-based vaccines. Inducing CD8^+^ T cell responses to conserved T cell epitopes has the ability to provide cross-protective immunity to evolving pathogens, which would otherwise escape neutralizing antibodies, such as SARS-CoV-2 (6, 7). Second, the ability to induce robust anti-tumor CD8^+^ T cells also positions their application in therapeutic cancer vaccines, as demonstrated in mouse studies (8–10) and recent phase I clinical trials (11, 12).

Prior studies have evaluated how innate immune induction coordinates with vaccine-elicited adaptive immune responses; however, many have been restricted to the study of peripheral blood in humans (13–16), with limited investigations in tissues in mice (17–21). These studies have not addressed the earliest kinetics across tissues. We, therefore, sought to elucidate the early spatiotemporal evolution of the immunological response following Ad vector vaccination. We aimed to integrate the early immune response with the induction of CD8^+^ T cell responses to understand the underlying factors that influence the immunogenicity of T cell-based vaccines.

We found that the initial wave of the immune response following intramuscular Ad26 vaccination commences by 1 hour and develops quickly over the first 24 hours across tissues and blood. Serum cytokines at 6 hours correlated with the frequency of vaccine-elicited CD8^+^ T cells at 60 days post-vaccination, suggesting that immunological events within the first few hours already have the potential to shape memory CD8^+^ T cell formation. These studies lay the foundation for more detailed mechanistic studies into vaccine-elicited innate immunity and its integration with ensuing adaptive immune responses.

## RESULTS

### The initial wave of immune response following Ad26 vector vaccination in mice occurs within the first 24 hours

**Fig 1:**
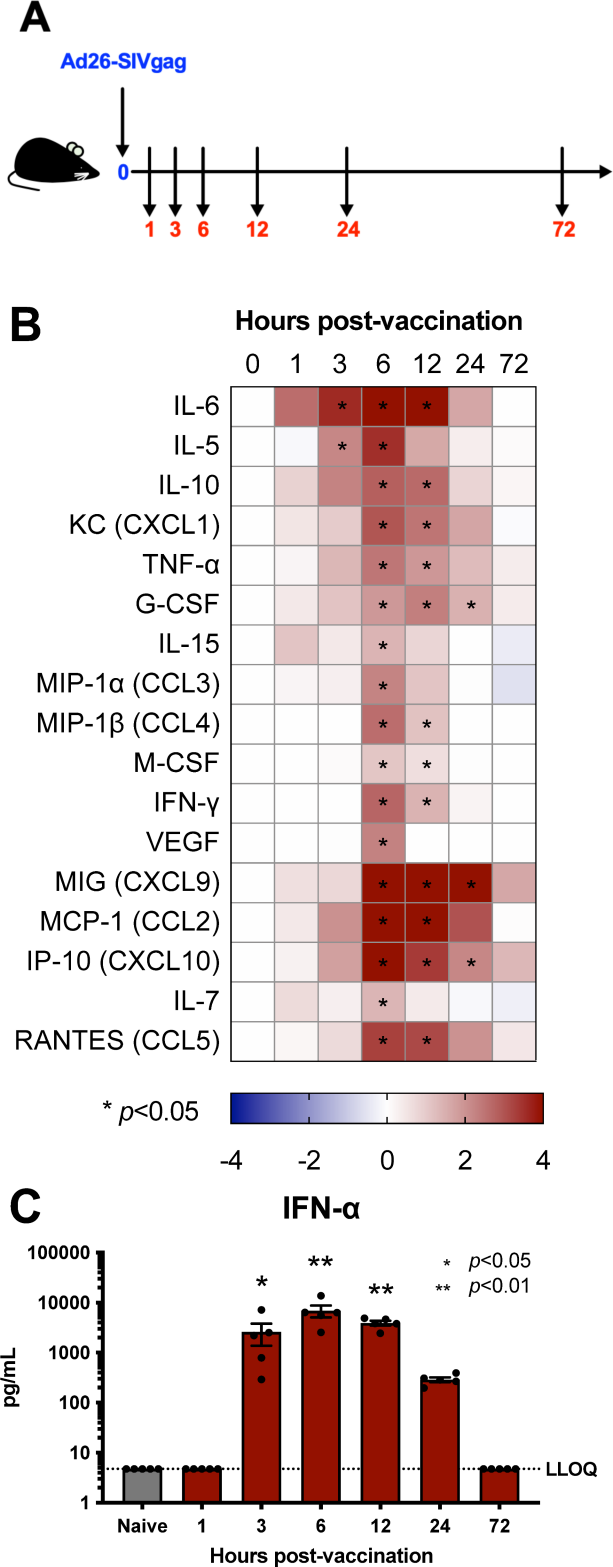
Intramuscular adenovirus serotype 26 vector vaccination rapidly induces serum cytokines, chemokines, and interferon. C57BL/6 mice were immunized intramuscularly with 1 × 10^10^ vp of Ad26-SIVgag. (**A**) Study outline, (**B**) cytokines and chemokines detected in serum via Luminex are shown as a heat map of the log2 fold change (LOG_2_FC) of the group average over the average naive reading, and (**C**) IFN-α levels as measured via IFN-α ELISA. N of 5 per group. Kruskal-Wallis test with Dunn’s corrections for multiple comparisons.

We first aimed to understand the general kinetics of the early immunological response following viral vector vaccination. C57BL/6 mice were vaccinated intramuscularly with a prototype Ad26 vaccine vector expressing SIVgag (Ad26-SIVgag), and we conducted a time course study focusing on the first 24 hours post-vaccination (Fig. 1A) at 1, 3, 6, 12, and 24 hours and an additional time point later at 72 hours to reflect the likely waning innate immune response. As cytokines and chemokines are critical for the initiation, coordination, and resolution of inflammation, we assessed the induction kinetics of cytokines and chemokines via multiplex bead-based ELISA (Luminex) assays in serum post-vaccination.

We found that the cytokine response initiates by significant IL-6 detection at 3 hours (*P* =< 0.05) (Fig. 1B). Peak responses occurred around the 6 hour time point (IL-6, IL-5, IL-10, IL-15, CXCL1, TNF-α, MIP-1α, MIP-1β, MIG, MCP-1, IP-10, IL-7, RANTES, and IFN-γ, all at least *P* =< 0.05) and a later set exhibiting a more prolonged detection at 24 hours (G-CSF, MIG, and IP-10, all at least *P* < 0.05). Similar kinetics were observed for IFN-α in serum (3–12 hours, all at least *P* =< 0.05) (Fig. 1C). Overall, responses began to wane by 24 hours and were mostly close to baseline by 72 hours (Fig. 1B and C). These data suggest that the initial cytokine and chemokine induction occurs in a rapid and transient fashion following intramuscular Ad26 vaccination, thus quickly coordinating the initiation and integration of immune responses.

### Ad26 vector vaccination results in rapid evolution of multiple immunologic pathways across blood and tissues within the first 24 hours post-vaccination

**Fig 2:**
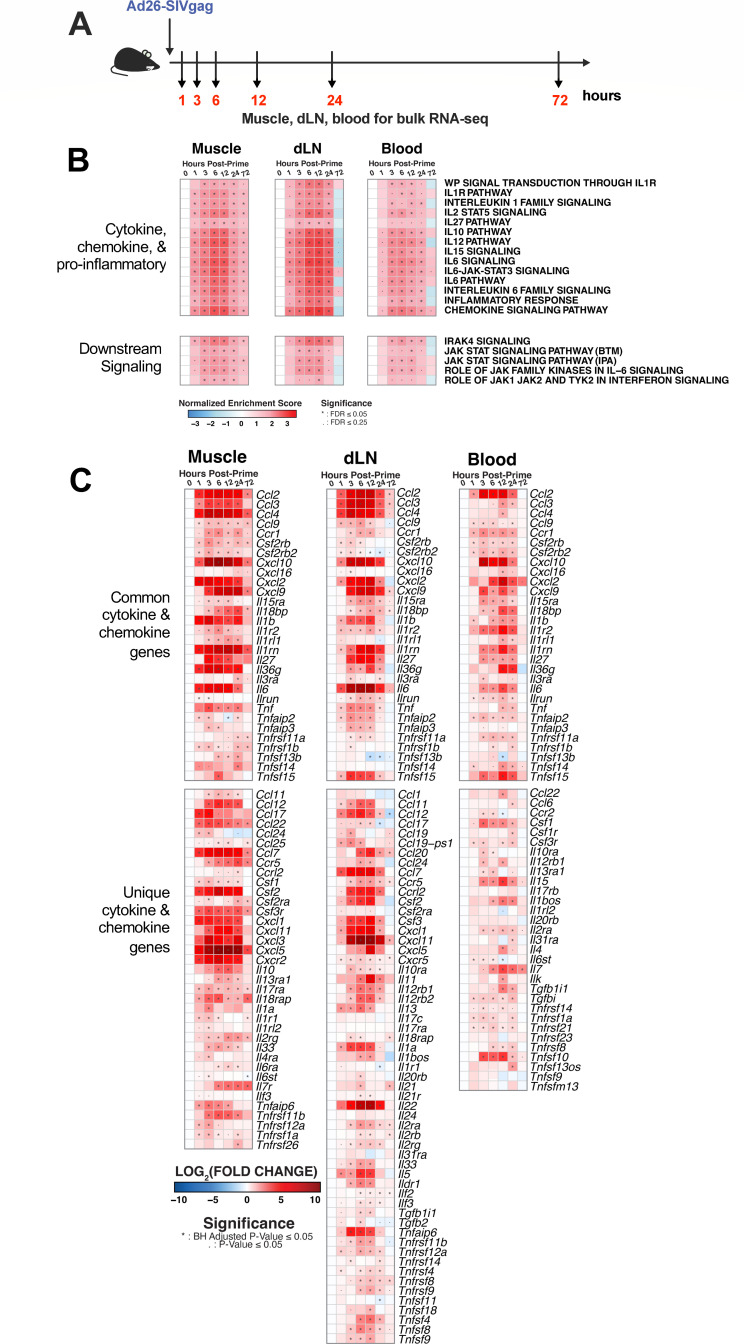
Ad26 vector vaccination broadly stimulates immunologic signaling pathways across tissues within the first few hours following vaccination. C57BL/6 mice were immunized intramuscularly with 1 × 10^10^ vp of Ad26-SIVgag, and samples were harvested for immune analysis via bulk RNA-seq across muscle, dLN, and blood. (**A**) Time course, (**B**) gene set enrichment analysis (GSEA) of immune signaling pathways, as measured by normalized enrichment score (NES) from naive, and (**C**) individual genes related to cytokines and chemokines (log_2_FC from naive). N of 5 per group.

We then sought to garner a global picture of how the immunological response to Ad26 vector vaccination develops across time and space in more detail. We collected blood and tissues to survey immune response kinetics by bulk RNA-seq transcriptomic profiling, including the site of vaccination in the muscle, the draining iliac lymph node (dLN) for priming of adaptive immune responses, and additionally peripheral blood (Fig. 2A). As cell-to-cell signaling is critical for initiating the coordination of immune responses, we first evaluated gene expression levels of cytokines and chemokines across all time points and compartments.

Across all three compartments, we observed rapid upregulation of a wide variety of pathways for cytokine and chemokine signaling and related downstream signaling (Fig. 2B, top and bottom panels, respectively). These included multiple pathways for proinflammatory IL-1 and IL-6 signaling, in addition to IL-12, IL-15, and chemokine signaling (Fig. 2B top panel, Tables S1 to S3). At 1 hour, we observed significant enrichment of these pathways in the muscle and the dLN compared to blood. These pathways were largely downregulated in the dLN and blood by 72 hours (Table S2), while still persisting in the muscle (IL-12 pathway *NES* = 1.73 *p*<0.0001, *FDR* < 0.001; IL-15 pathway *NES* = 1.67, *P* < 0.0001, *FDR* < 0.01; IL-6 signaling *NES* = 1.73 *p*<0.0001, *FDR* < 0.001) (Table S1). These data highlight the rapid induction of immune responses at the vaccine site and dLN, with following detectable responses in blood hours later.

We then evaluated individual cytokine and chemokine gene expressions. We observed a number of significantly upregulated genes that were shared across all three compartments with a general peak around 3–12 hours: *Ccl2, Ccl3, Ccl4, Ccl9, Cxcl10*, *Cxcl9*, *Il1b*, *Il6*, and *Tnf* (Fig. 2C top panel, Tables S4 to S6). While some degree of commonality existed across compartments, we observed clear differences in tissue-specific gene expression (Fig. 2C bottom panel). In the muscle, pro-inflammatory *Cxcl3* (3 hours *P* < 0.0001, 24 hours peak *P* < 0.0001) and anti-inflammatory *Ccl17* (1 hour *P* < 0.01, 3 hours *P* < 0.001), *Ccl22* (1 hour *P* < 0.001, 3 hours *P* < 0.0001, 6 hours *P* < 0.0001), and *Il10* (6 hours *P* < 0.01, 12 hours *P* < 0.001) were upregulated early, suggesting a balance of induced immune responses (22). Further, while the overall response waned by 72 hours, the muscle still exhibited prolonged expression of *Ccl2* (*P* < 0.0001), *Ccl4* (*P* < 0.01), *Cxcl10* (*P* < 0.001), *Cxcl9* (*P* < 0.01), *Ccl22* (*P* < 0.05), *Ccl7* (*P* < 0.0001), and *Cxcl5* (*P* < 0.05) (Fig. 2C; Table S4).

In the dLN, unique cytokines and chemokines related to the lymphocyte response and trafficking were marked by upregulation of *Ccl19* (3 hours *P* < 0.0001), *Ccl20* (6 hours *P* < 0.0001, 12 hours peak *P* < 0.0001), *Il11* (6 hours *P* < 0.01, 12 hours peak *P* < 0.0001), *Il13* (1 hour *P* < 0.01, 6 hours peak *P* < 0.01), *Il22* (3 hours *P* < 0.0001, 6–12 hours peak *P* < 0.0001), and *Il5* (1 hour *P* < 0.05, 6–12 hour peak *P* < 0.0001) (Fig. 2C bottom panel, Table S5). While in blood, the gene expression related to immune proliferation was uniquely elevated, as reflected by *Il15* (3 hours *P* < 0.0001, 12 hours peak *P* < 0.0002) and *Il7* (6 hours *P* < 0.0001, 12 hours peak *P* < 0.0001). Further, others trended toward a later peak response in blood compared to other compartments such as *Cxcl2* (12 hours *P* < 0.0001) and *Tnf* (12 hours *P* < 0.0001, 24 hours *P* < 0.01) and *Ccl22* at 12 hours (*P* < 0.05) in blood versus 1 hour (*P* < 0.0001) in the muscle (Fig. 2B bottom panel, Table S6).

When we considered integrative immune processes across compartments. We first observed more common chemokine genes upregulated between the muscle and dLN (Fig. 2C bottom panel). *Ccl7, Ccl12, Cxcl1, Cxcl11, and Cxcl5* are chemoattractants for lymphocytes and monocytes, together suggesting the initiation of monocyte trafficking and differentiation within hours in these two compartments. *Csf1* was upregulated between the muscle and blood, which has a role in stimulating the proliferation and differentiation of macrophages (Fig. 2C bottom panel). However, blood overall had fewer overlapping genes with either the muscle or dLN compartments. The overlap in the gene expression pattern between the muscle and draining lymph node could be reflective of the rapid spread of the immune response and coordination between the injection site and its draining lymph node.

**Fig 3:**
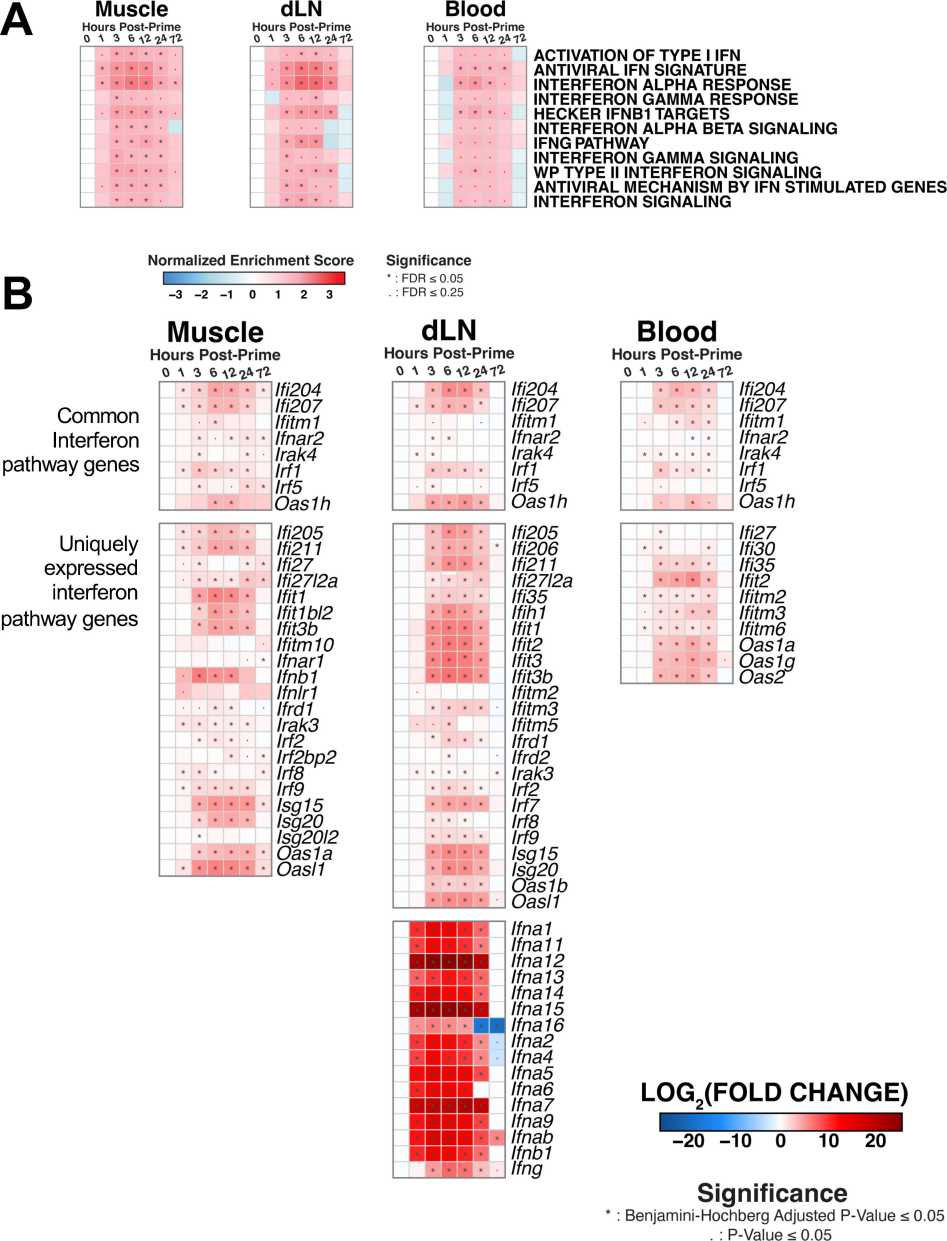
Interferon pathways are rapidly upregulated across tissues. Interferon responses across tissues were assessed by (A) GSEA of interferon signaling pathways measured by NES as compared to naive and (B) individual genes in the interferon signaling module, as measured by log_2_FC from naive. N of 5 per group.

Following our analysis of cytokine and chemokine signaling, we evaluated the enrichment of interferon family genes as type I interferon signaling has been shown to shape the development of T cell magnitude and polyfunctionality following vaccination with some Ad vector serotypes (23). We observed significant enrichment of many pathways associated with interferon responses and signaling across all compartments (Fig. 3A; Tables S1 to S3). Key downstream signaling genes *Irf9 (P < 0.0001*) and *Isg15 (P < 0.0001)* were upregulated by 3 hours in the muscle, dLN, and blood, alongside other associated genes. By initiating the response, IFN-α was produced only in the dLN as transcripts for multiple IFN-α subtypes were significantly induced starting at 1 hour: *Ifna1, Ifna2, Ifna4, Ifna5, Ifna6, Ifna7, Ifna9, Ifna11, Ifna12, Ifna13, Ifna14,* and *Ifna15* (all at least *P* < 0.05) (Fig. 3B bottom panel, Tables S3 to S6). Our data support and extend prior findings (13, 17, 23, 24) by showing that rapid production of IFN-α in the draining lymph node by 1 hour likely results in systemic upregulation of interferon pathways by 3 hours post-vaccination.

Together, these early pathway data suggest that immunological signaling pathways are significantly enriched not only at the injection site but also spreading to the draining lymph node as early as 1 hour post-Ad26 intramuscular vaccination, highlighting the rapid coordination of vaccine-induced immune responses. Furthermore, the patterns of overlap between the muscle and dLN, but to a lesser extent blood, suggest that blood alone may not fully capture the extent of immunological responses.

### Myeloid cells are early responders to Ad26 intramuscular vaccination

**Fig 4:**
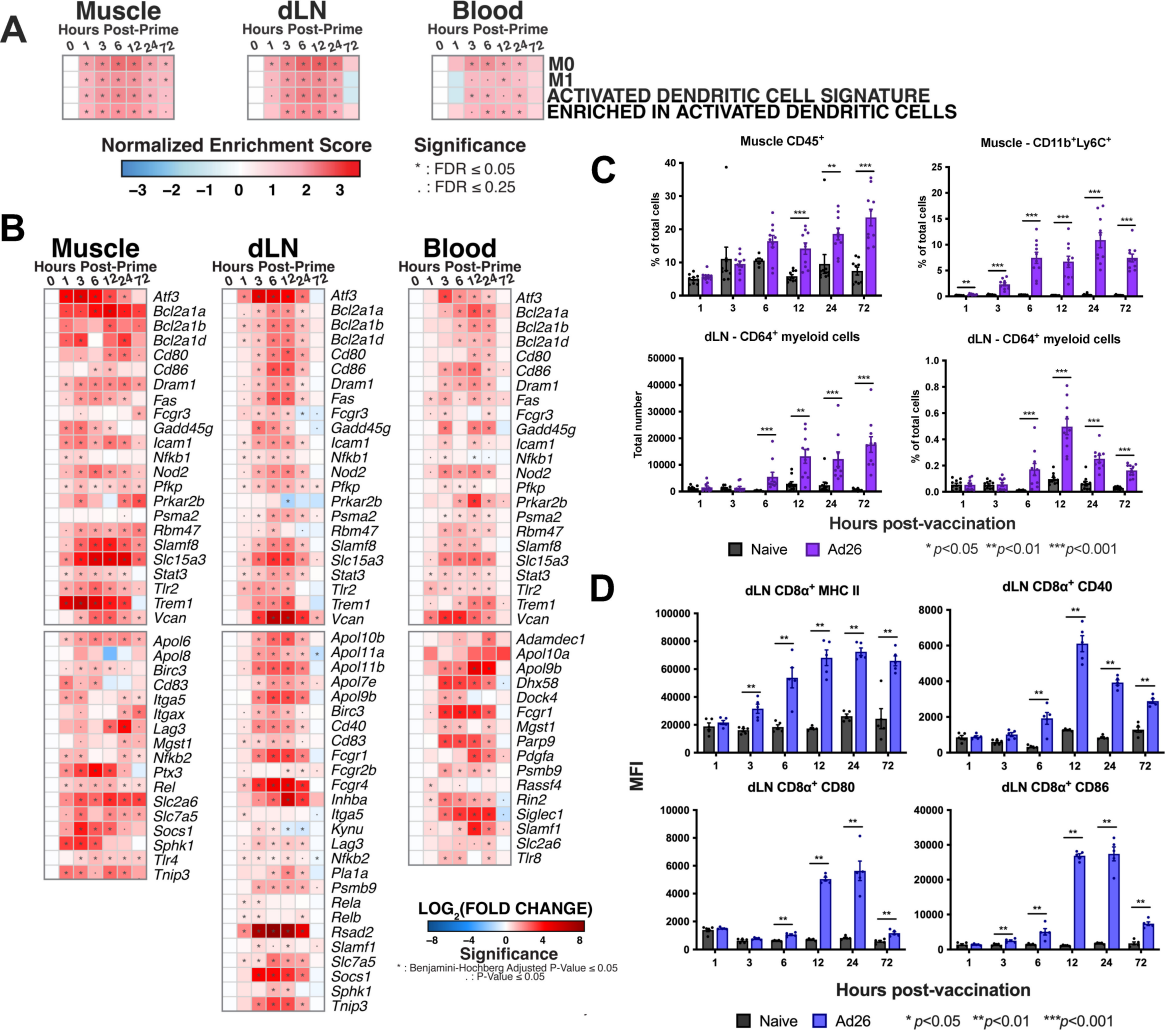
Early immunological responses are driven by myeloid cells. Bulk RNA-seq data were assessed for (A) immune cell signatures by GSEA and (B) related individual genes. To evaluate cellular responses via flow cytometry, C57BL/6 mice were immunized intramuscularly with 1 × 10^10^ vp of Ad26-SIVgag. Muscle, draining lymph node, and blood were collected, and immunological responses were profiled by (C) total frequency of CD45^+^ cells in the muscle, total frequency of CD11b^+^Ly6C^+^ myeloid cells in the muscle, total number of CD11b^+^Ly6C^+^CD64^+^ monocyte-derived dendritic cells in dLN, and total frequency of CD11b^+^Ly6C^+^CD64^+^ monocyte-derived dendritic cells in dLN. (D) Surface expression of MHC II (I-A/I-E), CD80, CD86, and CD40 on dLN CD8α^+^ dendritic cells, measured as the median fluorescence intensity (MFI). N of 5–10 per group, Mann–Whitney *U*-test.

Integrating our signaling data with cellular responses, we next sought to understand the immune cell components that could be initially driving and responding to the cytokine and chemokine signals by evaluating pathways for immune cell populations across all three compartments. M1 macrophage signatures were most consistently enriched post-vaccination with initial detection by 1 hour post-vaccination in the muscle (*NES* = 1.61, *FDR* < 0.05), followed by dLN at 3 hours (*NES* = 1.99, *FDR* < 0.001) and blood at 6 hours (*NES* = 1.66, *FDR* < 0.05) (Fig. 4A). We also observed rapid enrichment of the activated dendritic cell signature pathway in the muscle by 1 hour (*NES* = 1.71, *FDR* < 0.05), followed by 3 hours in dLN (*NES* = 2.02, *FDR* < 0.001) and blood (*NES* = 1.69, *FDR* < 0.05) (Fig. 4A).

Within the dLN at 6 hours, we observed continued enrichment of the activated dendritic cell pathway (*NES* = 2.26, *FDR* < 0.0001), with leading-edge genes *Irf7, Cd40, Cd80, Cd86, Ccl19, Cxcl10,* and *Cxcl11*, and the enriched in activated dendritic cells (NES = 2.08, *FDR* < 0.001) pathway, including genes *Il18, Il1b* (Fig. 4A and B; Table S5). These pathways suggest DC activation and maturation commencing within the dLN by 6 hours post-vaccination.

In order to confirm the transcriptomic changes that were observed post-vaccination, we profiled the response kinetics of myeloid cell populations. While the total frequency of CD45^+^ cells was not significantly higher at 1 hour post-vaccination in the muscle (Fig. 4C), we observed a significant increase in the frequency of CD11b^+^Ly6C^+^ immune cells at 1 hour post-vaccination (*P* < 0.01) continuing through to 72 hours (Fig. 4C), suggesting an accumulation of inflammatory monocytes. While trends emerged earlier, starting at 12 hours post-vaccination, we observed a progressive significant increase in CD45^+^ cells in the muscle (*P* < 0.001), reflecting increased immune cell recruitment to the initial injection (Fig. 4C). Together, these data indicate that while the total frequency of immune cells may not change significantly in the initial hours post-vaccination, changes occur within its composition. Of these immune cells, inflammatory monocytes are among the earliest responders at the vaccination site, followed by immune cell recruitment to the vaccine site.

We then considered the kinetics of immunologic responses in the dLN. We observed the appearance of a CD11b^+^Ly6C^+^CD64^+^ population in dLN starting by 6 hours post-vaccination (*P* < 0.001) (Fig. 4C). CD64 can be expressed on monocytes, macrophages, and monocyte-derived dendritic cells (mo-DC) (25). Previously published data have shown that antigen-carrying mo-DC were also found in the dLN 24 hours following subcutaneous vaccination with other Ad vector serotypes (17). The appearance of this population in the dLN occurred following detection of CD11b^+^Ly6C^+^ inflammatory monocytes in the muscle. Our data support prior findings and, by extension, suggest that Ly6C^+^ inflammatory monocytes and CD64^+^ myeloid cells may play a prominent and early role in the first few hours following intramuscular Ad26 vector vaccination.

It is known that dendritic cell cross-presentation of antigens is critical for the induction of CD8^+^ T cell responses following Ad vector vaccination, which includes the lymph node-resident CD8α^+^ DC population (17, 26). We, therefore, evaluated the response kinetics of the CD8α^+^ DC subset (CD11c^+^CD8^+^B220^-^). We observed a significantly increased expression of MHC II (I-A/I-E) first at 3 hours (*P* < 0.01) (Fig. 4D) and CD86 (*P* < 0.01), followed by co-stimulatory markers CD40 (*P* = 0.01) and CD80 (*P* = 0.01) at 6 hours post-vaccination, with reduced expression by 72 hours. Taken together, our studies showed rapid trafficking of myeloid cells into the muscle injection site within hours following vaccination. Considering the vast array of cytokines and chemokines that can be released by monocyte and macrophage populations, they may play a substantial role in promoting Ad26 the initial vaccine-elicited immune responses in the first few hours following intramuscular vaccination.

### CD8^+^ T cell immunogenicity can be shaped by 6 hours following Ad26 vaccination

**Fig 5:**
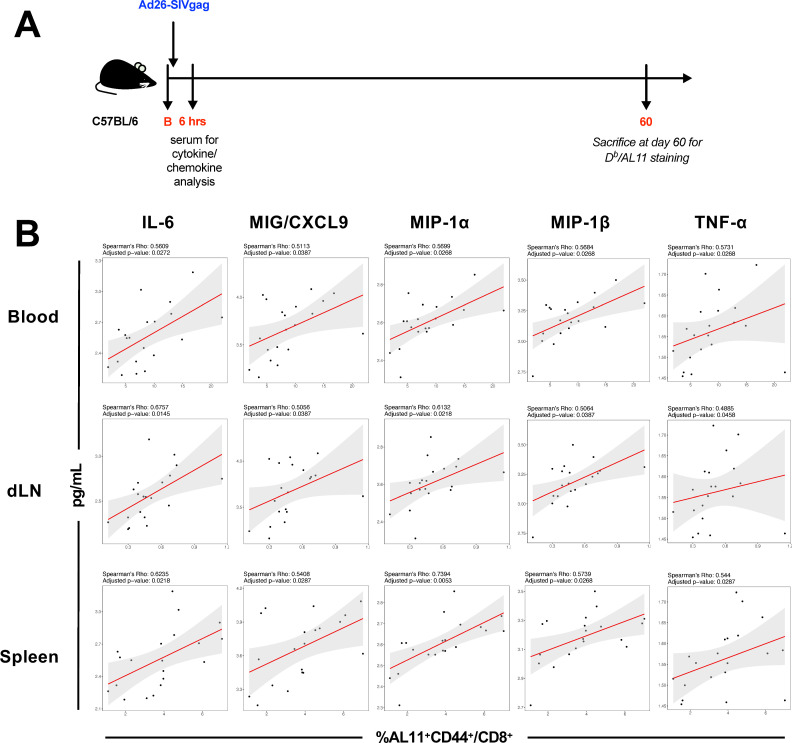
Serum cytokines at 6 hours post-vaccination are predictive of vaccine-elicited CD8^+^ T cell responses. C57BL/6 mice were immunized with 1 × 10^10^ vp of Ad26-SIVgag. Cytokine and chemokine protein levels were evaluated in serum via Luminex at 6 hours post-vaccination. (**A**) Outline of the study. (**B**) Individual cytokine correlation scatterplots of serum cytokine levels measured via Luminex with the frequency of SIVgag-specific CD8^+^ T cell responses measured via H-2D^b^ AL11 tetramer binding assays in blood, dLN, and spleen at 60 days post-vaccination. *N* = 20 mice per group. Correlations shown were calculated using a Spearman rank test, and *P*-values were adjusted for multiple comparisons using Benjamini–Hochberg corrections. Trend lines were calculated using a generalized linear model and are shown with 95% confidence intervals.

As innate immunity can shape and regulate adaptive immune responses, we sought to understand how markers of early immune responses could serve as an indicator of vaccine-elicited CD8^+^ T cell responses. We vaccinated mice with Ad26-SIVgag and collected the serum for protein-level cytokine analysis (Luminex) at 6 hours post-vaccination. We chose this time point as we previously observed the broadest degree of cytokine and chemokine detection in serum (Fig. 1B). Based on this earlier study, we studied nine cytokines and chemokines with direct effects on T cell responses that we also observed to be significantly induced following vaccination (IL-6, MIG/CXCL9, MIP-1α, MIP-1β, TNF-α, RANTES/CCL5, IFN-γ, IL-7, and IL-15). We evaluated the induction of SIVgag-specific CD8^+^ T cell responses via tetramer binding assays for the immunodominant SIVgag H-2D^b^ epitope, AL11 (27) at 60 days post-vaccination in blood and tissues (Fig. 5A). We found that for five of the nine selected cytokines and chemokines, the frequency of AL11-specific CD8^+^ T cells at 60 days post-vaccination in all three compartments positively correlated with the levels of IL-6 (*P* = 0.0272, *P* = 0.0145, and *P* = 0.0218, respectively), MIG/CXCL9 (*P* = 0.0387, *P* = 0.0387, and *P* = 0.0287), MIP-1α (*P* = 0.0268, *P* = 0.0218, and *P* = 0.0053), and MIP-1β (*P* = 0.0268, *P* = 0.0387, and *P* = 0. 0268), and TNF-α (*P* = 0.0268, *P* = 0.0458, and *P* = 0.0287) at 6 hours post-vaccination (Fig. 5B). Together, these data suggest that immunological events occurring by 6 hours post-vaccination may have the ability to shape the Ad26-vaccine-elicited CD8^+^ T cell response, including the generation of memory T cell responses.

## DISCUSSION

Adenovirus vectors have demonstrated their utility as vaccine platforms due to their ability to stimulate robust immune responses following vaccination. While much is known about immune responses elicited by Ad vectors, open questions remain as to what immediate immunological events occur following vaccination and in particular how these early events are unfolding in tissues that could potentially shape and regulate vaccine-elicited immunity.

Prior studies have investigated the rapidity of immune responses elicited by vaccination (17, 19–21, 28, 29). We hypothesized that we would observe immune responses unfold within hours following Ad26 vaccination, initiating from the site of vaccination, to the draining lymph node, and systemically reflected in blood. Building upon prior observations demonstrating Ad26-induced serum cytokine responses at 24 hours post-vaccination in non-human primates (24) and humans (30), we now use a mouse model to detail the evolution of the earliest immune responses during the first 24 hours post-vaccination across blood and tissues.

By transcriptomic analysis, enrichment of IL-1 and IL-6 pro-inflammatory pathways by 1 hour post-vaccination across tissues suggests a systemic rapid coordination of immune responses. These pro-inflammatory pathways likely initiate the cascade of increased cytokine and chemokine gene expression and protein levels at 3–12 hours. These responses typically peaked within the first 24 hours, indicating that while some degree of innate immune responses can be detected a day post-vaccination, earlier time points may be of more interest for surveying a greater breadth and magnitude of innate immune responses. Furthermore, the breadth of induced immune signaling pathways may suggest that Ad26 can broadly stimulate the induction of immune responses, which may contribute to its potent immunogenicity.

Some pathways exhibited commonality across anatomic compartments, suggesting key unifying immunological events. However, we also observed tissue-specific features. Innate immune responses waned quickly in blood but persisted in the muscle, likely due to the ongoing immune recruitment, as suggested by continued enrichment of myeloid cell gene signatures and detectable CD11b^+^LyC^+^ cells in the muscle at 72 hours. Additionally, overlap is observed more between the muscle and dLN in comparison to blood, suggesting that immune responses may be tightly coordinated between these two compartments. This also suggests that sampling of blood for the study of innate immune responses, while showing some unified immune responses, may not entirely reflect key immunological events that determine vaccine immunogenicity that are uniquely tissue-located.

When we evaluated potential serum biomarkers of vaccine immunogenicity, we observed that serum levels of IL-6, MIG/CXCL9, MIP-1α, MIP-1β, and TNF-α at 6 hours correlated with the frequency of SIV-gag AL11-specific CD8^+^ T cell responses in blood and tissues at 60 days post-vaccination. In hand, we observed *Il6*, *Cxcl9*, *Ccl3*, and *Ccl4* gene expressions in all three compartments, but more strongly upregulated in the muscle and dLN. IL-6 is a pleiotropic cytokine that has a role in various facets of pro-inflammatory immune responses and immune coordination. IL-6 has been shown to be produced rapidly by macrophages and dendritic cells following systemic intravenous administration of Ad5 (31). IL-6 has been shown to play a role in promoting Ad5 vaccine-induced CD8^+^ T cell responses as increased CD8 +T cell responses were observed following co-administration of Ad5 and IL-6 (8). Our data suggest a potential role of IL-6 in the coordination of Ad26 vaccine-elicited CD8^+^ T cell responses, which is likely multifactorial. Although the mechanism was not defined, MIP-1α (CCL3) has been shown to increase CD4^+^ T cell responses when encoded alongside vaccine antigens in an Ad5 vaccination mouse model (32). While we were able to identify potential serum biomarkers of vaccine immunogenicity, deeper mechanistic studies are warranted to understand the specific role of these cytokine and chemokine pathways in shaping Ad26 vaccine-elicited CD8^+^ T cell responses.

While we focused on Ad26, a prior study in mice evaluated transcriptomic responses at 8, 24, and 72 hours post-vaccination in the draining lymph node following subcutaneous vaccination with a variety of Ad vector serotypes including Ad5, Ad28, Ad35, chAd3, chAd63, sAd11, and sAd16 (17). In line with that study, we observe similar response kinetics in our data post-intramuscular Ad26 vaccination in the dLN. Another study analyzed early immune responses in the muscle and dLN following ChAd155 intramuscular vaccination in mice in which cytokine responses were detected at 1 hour in the muscle (21). This observation is concordant with findings with Ad26; however, we extend this knowledge by evaluating the broader immunologic transcriptomic networks involved in the immune cascade and integration of serum biomarkers with vaccine immunogenicity.

In our study, we used a mouse model due to the ease of tissue sampling to investigate the kinetics of tissue-specific immunity. Unlike its widely distributed expression in humans, CD46 expression in mice is limited to testes and retinal tissue. CD46 is a primary entry receptor for Ad26 (33). Prior studies investigating differences in T cell phenotypes with CD46 utilizing vectors have shown similarities between T cell responses in C57BL/6 and CD46 transgenic mice engineered to express the CD46 receptor, suggesting that the lack of CD46 does not dramatically impact the induction or shaping of vaccine-induced T cell responses in this mouse model (34). Intramuscular injection of Ad26 induces transgene expression in the muscle (35). These data suggest that regardless of the absence of CD46 expression, Ad26 can enter cells at the site of injection in the muscle and as such potentially may use alternative entry mechanisms in this vaccination route in mice.

While we have not explored the earliest kinetics and potential contributions of transgene expression in this present study, our group has previously shown that the vector in the absence of the encoded transgene also triggers innate immune responses (36). Adenoviral vector trafficking within endosomes and triggering of TLR9 and other innate immune pathways occur prior to nuclear localization (36), and thus initial stimulation of innate immune responses by the Ad vector is more likely a factor of the vector itself more than the transgene-encoded vaccine antigen. However, additional studies will be required to define if the transgene contributes to innate responses.

Our study design used bulk RNA-seq to survey a large number of samples across tissues and time points. A limitation of this approach is that bulk RNA-seq cannot capture immune cell heterogeneity and specific functional assignment on a per-cell basis. Myeloid cell populations can differentiate into a variety of states and subsets in the context of inflammation; thus, minor and novel subsets cannot be defined through this approach. Moving forward, studies utilizing single-cell approaches will provide greater depth of immunological cell states and their corresponding functional signatures.

Taken together, our data show that the innate immune response elicited by Ad26 vaccination commences by 1 hour post-vaccination and rapidly evolves within the first 24 hours across the site of vaccination, the draining lymph node, and blood. Immunologic pathways suggest rapid coordination of immune responses, immune cell trafficking, and cellular responses. While CD8α^+^ cross-presenting DCs are critical for the induction of CD8^+^ T cell immunity, the monocyte/macrophage lineage may be a significant contributor to initiating immune responses following intramuscular Ad26 vector vaccination. Furthermore, correlation data suggest that immunological events occurring within a few hours post-vaccination may shape the vaccine-elicited memory CD8^+^ T cell response. These data highlight the rapidity of the innate immune system in tissues in initiating and shaping the ensuing vaccine-elicited adaptive immune response and merit deeper investigation into early mechanisms of immune induction for understanding rational vaccine design. Future studies should also define the early spatiotemporal evaluation of innate and adaptive immune responses with other vaccine platforms.

## MATERIALS AND METHODS

### Immunizations

Female C57BL/6 mice were purchased from Jackson Laboratories (Bar Harbor, ME). Replication-incompetent, recombinant E1/E3-deleted adenovirus serotype 26 (Ad26) vectors were previously constructed (34, 37). Mice were immunized by bilateral intramuscular injection into the hind leg quadriceps with 10^10^ viral particles (vp) per mouse.

We performed a dose-titration experiment to determine the optimal dose for investigating innate immune responses. C57BL/6 mice were vaccinated intramuscularly into the hind leg quadriceps with escalating doses of an Ad26 vector expressing SIVgag: 1 × 10^8^, 1 × 10^9^, and 1 × 10^10^ viral particles (vp). We observed that at 8 hours post-vaccination, some cytokines were below the limit of detection at a dose lower than 1 × 10^10^ vp (unpublished). This suggests immune responses may be low and potentially below assay detection limits depending on the vector dose. Therefore, for these studies, we used a 1 × 10^10^ vp dose for evaluating innate immune responses in order to better detect low-level immune responses.

### Transcriptomic analyses

Tissue samples were collected into RNAlater (Invitrogen). Tissue samples were then transferred to QIAzol and homogenized with a TissueLyzer using 5 mm steel beads (all Qiagen). Blood was processed as outlined in the sample collection section, with cell pellets resuspended in QIAzol. Total RNA was extracted according to the QIAcube HT RNA extraction protocol (Qiagen). The Dana-Farber Molecular Biology Core Facility evaluated RNA quality via the Agilent 2100 Bioanalyzer (Agilent Technologies) and prepared RNA-seq libraries. Single-end 75 bp libraries were barcoded for multiplexing and sequenced with 20,000 reads per sample on an Illumina NextSeq 500.

#### RNA-seq analysis

All operations were performed locally, in R (version 4.3.1) (38). To slightly reduce noise, raw RNA counts were filtered such that genes with counts greater than 0 across all animals were preserved for further analysis. Initial principal component analysis (PCA) and data visualization were performed on normalized counts, using the “plotPCA()” function in R’s DESeq2 (version 1.40.2) (39). Due to the robust clustering observed in the PCA, where the first principal component (PC1) explained approximately 80% of the variance, each tissue was analyzed separately. This approach allowed us to focus on tissue-specific responses without being confounded by inter-tissue variability. Counts were then normalized using the “deseq()” function. Differential gene expression was computed for post-vaccination time points by contrasting each time point to baseline/pre-vaccination with the “results()” function in DESeq2 (39). Parameters in DESeq2 were left to default.

To assess pathway activity, differentially expressed genes were ranked in decreasing order by their log fold-change compared to baseline. To ascertain whether our fold changes were within biological plausibility, we repeated differential gene expression using shrunken log-FCs (using DESeq2’s lfcShrink (39) and again by first eliminating genes whose raw counts totaled 0 across all animals at baseline. Both methods yielded concordant results with our initial analysis.

This ranked list was then input to GSEA Pre-Ranked (version 4.2.3) using a pre-compiled set of pathways as our reference gene set database and default parameters (40, 41). To focus on pathways upregulated early post-prime, those with a significant upregulation (i.e., a false discovery rate (FDR) ≤ 0.25) in hours 1, 3, and 6 were plotted as a time course for each tissue, using ggplot2 (version 3.4.4) in R (42).

Further, to determine leading-edge genes for each pathway (for each tissue, at each timepoint), we used GSEA’s leading-edge tool on our previously computed GSEA outputs (41). Then, to resolve early gene expression behaviors, leading-edge genes from pathways of interest were plotted in terms of their log fold-changes at 1 hour post-prime (42).

### Sample collection for immunologic studies

Blood was collected into RPMI 1640 media (Corning) containing 5 mM of EDTA (Life Technologies). Lymphocytes were isolated using Ficoll–Hypaque (GE Healthcare) density centrifugation. The interphase was collected into R10 media, washed, and isolated cells were then used for subsequent assays.

For early time course studies, muscle and draining lymph nodes were collected into R5 media (RPMI (Corning), 5% FBS (Sigma), and 1% Pen/Strep (Life Technologies)). Tissue samples were cut into pieces and placed into R5 media containing collagenase Type IV (Sigma) and then digested for 1 hour at 37°C on a rocker. Following digestion, samples were passed through a 70 µm filter, and any remaining pieces were ground and washed through the filter with R5. All samples were washed once and resuspended in R10 media (RPMI, 10% FBS, 1% Pen/Strep) containing benzonase (Millipore).

For evaluation of day 60 T cell responses via tetramer staining, collected tissues were harvested and collected into R10 media (RPMI (Corning), 10% FBS (Sigma), and 2% pen/strep (Life Technologies). Spleen and draining lymph node samples were ground through 70 µm filters. Spleen samples were treated once with 1X ACK lysis buffer to remove red blood cells. All samples were washed with R10 and passed through a 30 µm filter. Samples were resuspended in R10 media containing benzonase (Millipore).

### Cytokine and chemokine assays

Frozen serum samples were thawed on ice and subsequently centrifuged for 10 minutes at 10,000 rpm. Serum was treated with 0.05% Tween-20 (Sigma) in 1X DPBS (Life Technologies) for 15 minutes at room temperature. Cytokine and chemokine levels were assessed using the Milliplex Mouse 32-plex premix kit (Millipore) as per the manufacturers’ instructions. Samples were subsequently fixed with 2% formaldehyde in 1X DPBS (Life Technologies) for 1 hour at room temperature. Following this, samples were washed, resuspended in Drive Fluid (Luminex Corp.), and run on a Magpix with Xponent software (Luminex Corp). Data were analyzed using a 5-parameter logistic model with an 80%–120% standard acceptance range. Extrapolated data below the limit of quantification were graphed and analyzed at the lower limit of quantification for the specific analyte.

### Flow cytometry

Single-cell suspensions were first stained with Fixable Blue or Near-IR vital dye in 1X DPS (Life Technologies) for 20 minutes at 4°C. Samples were subsequently washed, blocked with Fc block (TruStain FcX PLUS anti-CD16/CD32, Biolegend) and monocyte block (True-stain monocyte blocker, Biolegend) at 4°C for 15 minutes, and then stained with surface antibodies in MACS buffer (MACS wash buffer (Miltenyi Biotec), BSA (Miltenyi Biotec), Pen/Strep (Life Technologies)), and Brilliant Stain Buffer Plus (BD Biosciences) for 60 minutes at 4°C. For innate profiling experiments, antibody panels included the following: CD45 (clone 30-F11), B220 (RA3-6B2), CD8a (53–6.7), CD80 (16–10A1), CD86 (GL1), CD11c (N418), CD19 (clone 6D5), CD3 (clone 145–2C11), NK1.1 (PK136), MHC II (M5/114.15.2), CD64 (X54-5/7.1), CD40 (3/23), Ly6C (AL21), CD11b (M1/70), and Ly6G (1A8). For tetramer staining experiments, antibodies included the following: CD8a (53–6.7), CD44 (IM7), and AL11 tetramer. AL11 monomers were provided by the NIH tetramer core facility (Emory University, Atlanta, GA) and tetramerized using streptavidin conjugated to Brilliant Violet 421 (Biolegend). All antibodies were obtained from Biolegend or BD Biosciences. Following staining, samples were washed and fixed with 2% formaldehyde. Data were acquired on a FACSymphony (BD Biosciences) or LSR II (BD Biosciences) using BD Diva software and analyzed using FlowJo v10 (Treestar).

### Statistics

Statistical analyses on immunologic data in Fig. 1 to 4 were performed using GraphPad Prism 7, using tests as indicated in the text and corrected for multiple comparisons where indicated. Analyses in Fig. 5 were performed in R using the psych (43) and corrplot (44) packages to, respectively, compute and visualize correlations between serum levels of nine cytokines and chemokines (IL-6, MIG/CXCL9, MIP-1α, MIP-1β, TNF-α, RANTES/CCL5, IFN-γ, IL-7, and IL-15) and the frequency of SIV-gag AL11-specific CD8^+^ T cell responses in blood and tissues at 60 days post-vaccination. More specifically, the corr.test function was used to perform Spearman correlations between cytokines and T cell responses across our three tissues, and adjusted, and the false discovery rate was corrected with Benjamini–Hochberg multiple comparison adjustments (43). A correlogram from these results was plotted using the corrplot package, and correlations that were determined to be significant after adjustments were denoted by one to three stars, as shown in the figure legend. Individual cytokine correlations were presented in scatterplots using ggplot2 (42). For qualitative purposes, a trendline was displayed with 95% confidence intervals using the geom_smooth function with the following parameters: method = “glm,” formula = y ~ x, se = TRUE.

## Data Availability

Raw data for bulk RNA-seq studies are available in GEO under accession number GSE264344. All other data that support the findings of this study are available from the corresponding author upon request.

## SUPPLEMENTAL MATERIAL

Table S1 jvi.00247-25-s0001.xlsx GSEA muscle.

Table S2 jvi.00247-25-s0002.xlsx GSEA dLN.

Table S3 jvi.00247-25-s0003.xlsx GSEA blood.

Table S4 jvi.00247-25-s0004.xlsx DEG muscle.

Table S5 jvi.00247-25-s0005.xlsx DEG dLN.

Table S6 jvi.00247-25-s0006.xlsx DEG blood.

ASM does not own the copyrights to Supplemental Material that may be linked to, or accessed through, an article. The authors have granted ASM a non-exclusive, world-wide license to publish the Supplemental Material files. Please contact the corresponding author directly for reuse.
